# Elaboration and controlling excited state double proton transfer mechanism of 2,5-bis(benzoxazol-2-yl)thiophene-3,4-diol

**DOI:** 10.1038/srep44897

**Published:** 2017-03-22

**Authors:** Jinfeng Zhao, Yujun Zheng

**Affiliations:** 1 School of Physics, Shandong University, Jinan 250100, China

## Abstract

In the present work, we theoretically illuminate the excited state double proton transfer (ESDPT) process about a novel synthesized system 2,5-bis(benzoxazol-2-yl)thiophene-3,4-diol (BBTD). Minor changes of both structure and charge redistribution deriving from photoexcitation result in obviously different excited state dynamical process. Exploration about our constructed S_1_-state potential energy surface (PES) indicates a stepwise ESDPT mechanism for BBTD. In addition, we present a new mechanism about regulating and controlling stepwise ESDPT process via external electric field.

Proton transfer, as a kind of site-specific interaction through hydrogen bond, is one of the most significant reactions in chemical and biological acid-base dynamics^1^,^2^,^3^,^4^. Excited state proton transfer (ESPT) is a photo-tautomerization process occurring in the electronically excited state, which has been receiving considerable attention^5^,^6^,^7^. Most ESPT processes refer to the single proton transfer. In general, upon the photoexcitation, proton transfer process occurs along with an intra- or inter- molecular hydrogen bond between proton acceptor and proton donor in the low excited state. Basically, this kind of transfer process starts from the ground (enol) state and returns to the same state following a four-level reaction cycle. The fundamental process can be summarized as follow: On account of excitation, the molecule can be projected on a potential energy surface making the position of a proton unstable. Due to the energy gap between local excited state and relaxed excited state, the driving force is provided for the transformation. Meanwhile, the slope of the surface connecting these two points offers relative kinetics. After proton transfer reaction, the phototautomer (keto*) (i.e. the proton-transfer structure) form emits a longer wavelength fluorescence (the most significant Stocks shift can be as large as 6000–12000 cm^−1^)^8^,^9^,^10^. The relaxation of keto* results in the ground-state keto structure that undergoes a reverse ground state proton transfer back to enol species. It is the primary changes about electronic structures and charge distributions under the photoexcitation, ESPT processes own obviously different photophysical and photochemical properties providing various applications, such as chemical sensing^11^, fluorescence probes^12^,^13^, while light LED^14^, cell imaging, and so forth^15^,^16^,^17^.

In fact, most of biological systems refer to multiple protons transfer reaction. Only single proton transfer process is not enough to simulate biological processes. Particularly, the migration of protons along with a series of proton relays bridged through hydrogen bonded wires^18^,^19^,^20^. In order to explore properties of excited state multiple protons transfer, we believe that the investigation about excited state double proton transfer (ESDPT) reaction is the most fundamental way. Recent years, double inter- or intra- molecular hydrogen bonds have attracted much attention in interdisciplinary fields such as biology and material science. Barbatti *et al*. investigated the ESDPT process of 7-azaindole dimer in gas phase^21^. Zhao *et al*. studied proton transfer processes of bis-2,5-(2-benzoxazolyl)-hydroquinone as well as its derivatives and provided a competitive mechanism between S_1_-state single and double proton transfer processes^22^. Zhang *et al*. reported a detailed theoretical investigations about pigment yellow 101 on its excited state single or double proton transfer process^23^. As a whole, the investigations of double proton transfer process are very important, which could help researchers to have a deeper understanding about multiple protons transfer mechanism.

In addition, due to the photophysical and photochemical properties of ESIPT product (i.e. the keto* form), the regulation and control of ESIPT reaction has also caused extensive attention. Tomin *et al*. mentioned that excited state single proton transfer process might be affected by solvent polarity^6^. Banerjee *et al*. reported that the confined media also largely have effect on ESIPT process^24^. And the most common in experiment is that PH controls excited state dynamical process. In effect, electric field effects are of considerable interest in exploring biological environments^25^,^26^,^27^. In 4′-N,N-(diethylamino)-3-hydroxyflavone (DEHF) molecule, Klymchenko *et al*. studied electric field effect on ESIPT reaction and found apparent variation about intensity of its fluorescence^28^.

Recently, a new system 2,5-bis(benzoxazol-2-yl)thiophene-3,4-diol (BBTD) including two intramolecular hydrogen bonds is designed and synthesized^29^. As a model compound, Chen *et al*. use BBTD to explore single or double proton transfer process in the S_1_ state. Measuring absorption and emission spectra of BBTD, they find two large Stokes shifted fluorescence bands, which are attributed to BBTD-SPT and BBTD-DPT configurations (shown in Fig. 1), respectively. Moreover, considering the fluorescence decay of BBTD, they ensure excited-state single or double proton transfer process existing in the S_1_ state.

In effect, the explicit mechanism (stepwise or synchronous) about the ESDPT process is missing for BBTD system in previous work^29^. It is understandable that spectroscopic techniques such as absorption and emission spectra, time-resolved fluorescence spectroscopy, and so on, could only provide some indirect information about photophysical or photochemical properties^30^,^31^,^32^,^33^. In this work, to provide a clear and detailed ESDPT overall perspective, we theoretically study the excited state dynamical process of BBTD using density functional theory (DFT) and time-dependent DFT (TDDFT) methods^34^,^35^,^36^,^37^. We confirm a stepwise ESDPT process for BBTD. To the best of our knowledge, in addition, no study has reported on electric field effects on ESDPT reaction. We present a new mechanism about regulating and controlling ESDPT reaction via external electric field effect for the first time.

Our paper is organized as follows. Initially, we describe details of the calculations. Then the following section describes and discusses the results, which is organized by subsections consider electronic spectra, geometric structures, frontier molecular orbitals (MOs) and lastly potential energy surfaces. A final section summarizes and gives the conclusions of this study.

## Computational Details

In this work, all the quantum chemical calculations are mainly accomplished based on the density functional theory (DFT) and time-dependent density functional theory (TDDFT) methods with Beckes three-parameter hybrid exchange function with the Lee-Yang-Parr gradient-corrected correlation functional (B3LYP)^38^ in combination with the triple-ζ valence quality with one set of polarisation functions (TZVP)^39^ basis set by Gaussian 09 programs^40^. To be consistent with the previous experiment^29^, chloroform solvent is selected based on the Polarizable Continuum Model (PCM) using the integral equation formalism variant (IEFPCM)^41^,^42^, which considers the solute in a cavity of overlapping solvent (with an average area of 0.4 Å^2^) that has apparent changes to reproduce the electrostatic potential due to the polarized dielectric within the cavity. All the geometries of S_0_ and S_1_ states are optimized without constrains of bond lengths, bond angles and dihedral angles except for additional remarks, and vibrational frequency calculations are analyzed to confirm all the related structures corresponded to the local minima on the S_0_ and S_1_ potential energy surfaces (PESs). Zero-point energy corrections and thermal corrections to the Gibbs free energy are also performed according to the harmonic vibrational frequencies. The thermal correction to Gibbs free energies of all the stable structures are shown in Table S1, ESI†. Vertical excitation energy calculations are performed from the S_0_-state optimized structure using TDDFT method with six low-lying absorption transitions. In addition, we construct S_0_ and S_1_ PESs to further illustrate the ESDPT mechanism of BBTD system. All the stationary points along the reaction coordinate are scanned by constraining optimizations and frequency analyses (no imaginary frequency) to obtain the thermodynamic corrections in the corresponding electronic state.

## Results and Discussion

### Structures and MOs

The six low-lying absorbing transitions and fluorescence of BBTD, BBTD-SPT and BBTD-DPT structures are calculated (see Fig. 2). Our calculated absorption and fluorescence peaks of BBTD are 397 nm and 440 nm, respectively, which are consistent with experimental results (394 nm and 430 nm)^29^. In addition, our fluorescence peaks for BBTD-SPT and BBTD-DPT are 499 nm and 560 nm, they are also in line with the experimental results (475 nm and 550 nm)^29^, respectively. Herein, we confirm adequately the accuracy of the theoretical methods we adopted in this work.

**Figure 1:**
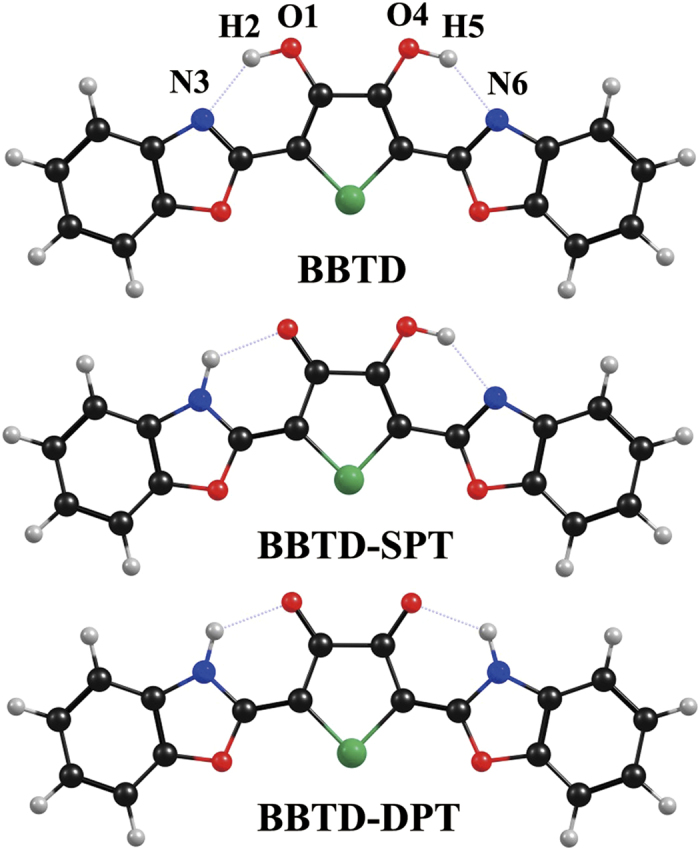
Views of BBTD, BBTD-SPT (single-proton transfer BBTD) and BBTD-DPT (double-proton transfer BBTD). Atom labels involved in two intramolecular hydrogen bonds are marked on BBTD structure. Red: O atom; Blue: N atom; Black: C atom; Green: S atom.

**Figure 2:**
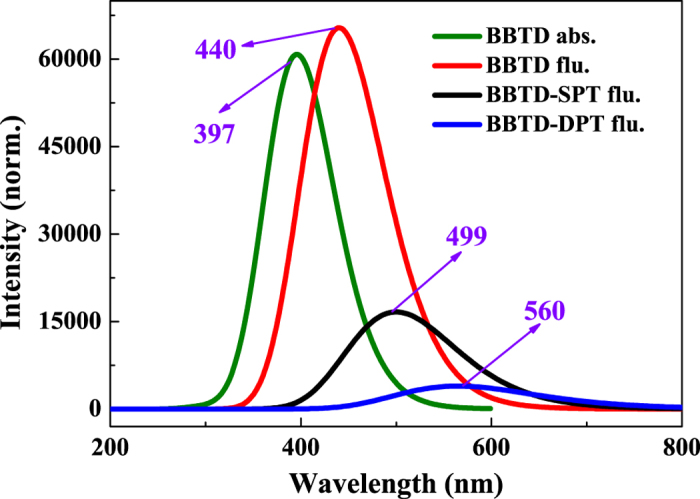
The theoretical electronic spectra of BBTD, BBTD-SPT and BBTD-DPT structures.

The structures of BBTD (normal BBTD), BBTD-SPT (single-proton transfer BBTD) and BBTD-DPT (dual-proton transfer BBTD) (shown in Fig. 1) are obtained within the framework of DFT and TDDFT methods as mentioned above, with a subsequent vibrational frequency analysis to insure the validity of the stationary points. We list some significant parameters involved in these two intramolecular hydrogen bonds (O_1_-H_2_ 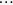 N_3_ and O_4_-H_5_ 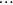 N_6_) in Table 1. Obviously, for BBTD structure, both O_1_-H_2_ and O_4_-H_5_ are elongated in the S_1_ state, whereas hydrogen bonds H_2_ 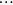 N_3_ and H_5_ 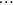 N_6_ are shortened with the concomitant enlargement of bond angle δ(O_1_-H_2_ 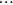 N_3_) and δ(O_4_-H_5_ 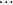 N_6_). Thus these two intramolecular hydrogen bonds are strengthened upon the photoexcitation^3^. Further, monitoring the infrared (IR) vibrational spectral shifts^3^, as another effective way to verify the changes about excited state hydrogen bond, is also adopted in this work. We show the vibrational spectra of BBTD form in the conjunct vibrational region of both O_1_-H_2_ and O_4_-H_5_ stretching modes in Fig. S1, ESI†. It is worth mentioning that red shift from S_0_ to S_1_ is around 12 cm^−1^, which is ascribed to the enhanced effect of excited-state hydrogen bonds (O_1_-H_2_ 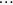 N_3_ and O_4_-H_5_ 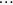 N_6_). Even though extent of variation of both bond lengths and bond angles is not big, it can result in important excited state dynamical process.

**Figure 3:**
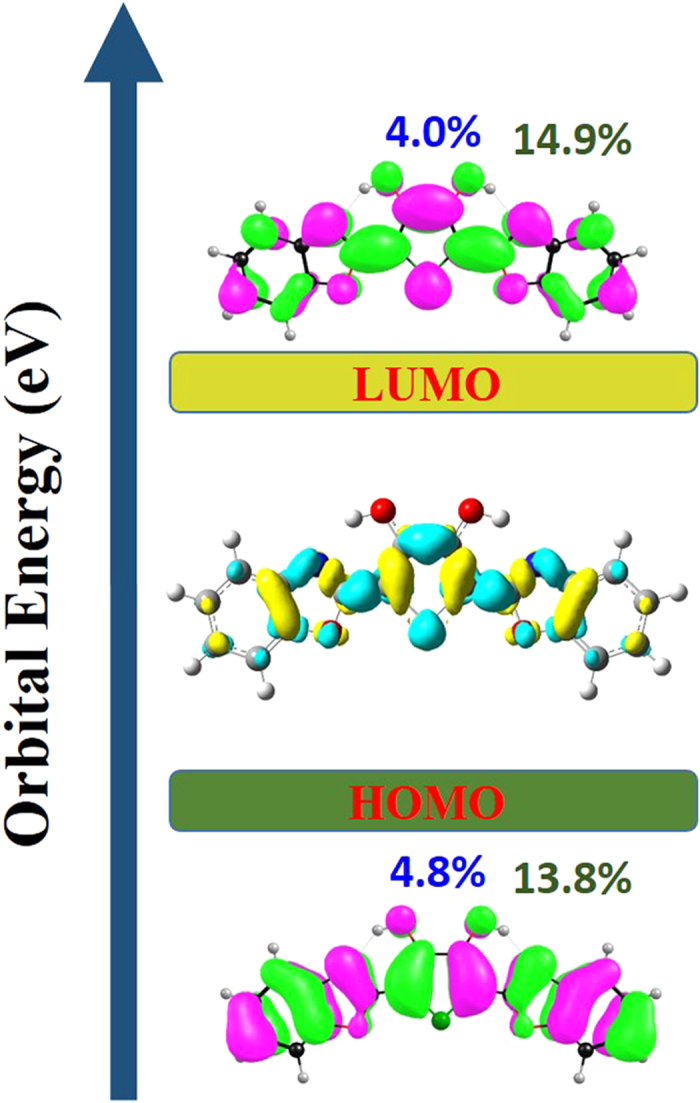
View of frontier molecular orbitals (HOMO and LUMO) for BBTD system. Herein, pink moiety means positive charge distribution and green moiety means negative charge distribution. Electron-density difference (EDD) map are shown between S_1_ and S_0_. In the EDD map, the regions with increasing electron density from S_0_ to S_1_ state are shown in cyan, and those with decreasing electron density are shown in yellow.

In addition, it is well known that charge redistribution stemming from photo-excitation could depict qualitatively the corresponding properties of electronically excited state. In this work, we show the frontier molecular orbitals (MOs) of BBTD molecule in Fig. 3. Since the S_1_ state of BBTD mainly refer to the highest occupied molecular orbital (HOMO) and the lowest unoccupied molecular orbital (LUMO) with a large oscillator strength (1.466), thus we only provide these two orbitals in this figure. Obviously, BBTD is a ππ*-type transition. Since the changes of charge distribution are not obvious, quantificational contributions above primary atoms involved in hydrogen bonds (O_1_-H_2_ 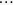 N_3_ and O_4_-H_5_ 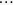 N_6_) are also calculated. The contribution of both O_1_ and O_4_ atoms drops from 4.8% (HOMO) to 4.0% (LUMO), where that of N_3_ and N_6_ increases from 13.8% to 14.9%. In addition, to be more visual, the electron-density difference (EDD) map are also calculated shown between HOMO and LUMO orbital in Fig. 3. The EDD map displays that upon excitation from S_0_ to S_1_ state net electron densities shift from hydroxyl groups to N_3_ and N_6_ moieties. It suggests that after the excitation a driving force can be induced to facilitate the proton transfer reaction in the S_1_ state. This is consistent with the physical picture obtained from the HOMO-LUMO transition. In effect, these minor variations of charge distributions reveal the tendency of ESDPT and provide the possibility for ESDPT process.

### Analysis of mechanism

**Figure 4:**
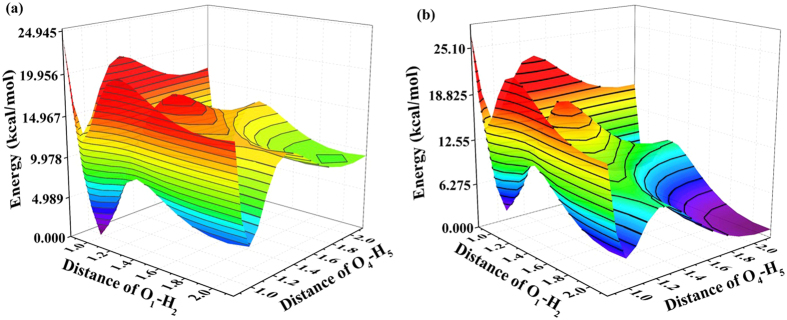
The S_0_-state (**a**) and S_1_-state (**b**) PESs of BBTD system as functions of O_1_-H_2_ and O_4_-H_5_ bond lengths.

**Figure 5:**
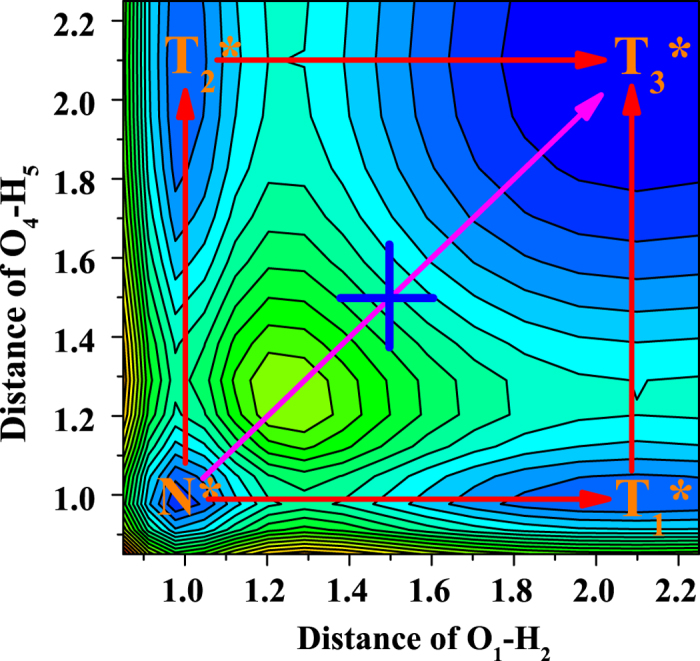
The S_1_-state projective plane with four stable points (N*, 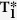, 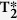 and 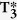) . The energy between every contour is 1.305 kcal/mol.

To explore specific ESDPT mechanism of BBTD system, we construct the S_0_-state and S_1_-state PESs as functions of both O_1_-H_2_ and O_4_-H_5_ bond distances from 0.85 to 2.25 Å (shown in Fig. 4). In this range, all the relative structures (BBTD, BBTD-SPT and BBTD-DPT) could be included. For convenience narration, we separate the S_1_-state projective plane in Fig. 5. It can be clearly found that four stable structures exist in this PES (i.e. N* point stands for BBTD form; 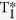 and 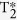 point stand for BBTD-SPT form; 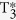 point stands for BBTD-DPT form). Due to the symmetry of BBTD, the PES is symmetrical along with diagonal line. That is to say, 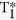 point and 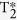 point correspond to the same structure (BBTD-SPT). Since some previous papers have demonstrated that TDDFT method can relatively accurate excited state pathways^43^,^44^,^45^, the potential energy barriers among these four stable configurations are calculated at TDDFT/B3LYP/TZVP level. In fact, to check the level of B3LYP is appropriate to describe this system, the results of potential energy curves are compared between B3LYP functional and a long-range corrected functional (i.e. Cam-B3LYP^46^) (see Fig. S2, ESI†). It can be clearly found that the trend of potential energy curves are consistent under these two functionals and the barriers are almost the same, which confirms the feasibility of B3LYP functional adopted in this work. Our results show that a 7.21 kcal/mol potential barrier separates N* point from 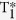 or 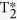 point, and a low barrier (4.58 kcal/mol) is needed to cross from point 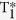 or 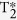 to point 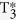. However, a high potential barrier (14.3 kcal/mol) separates point N* and point 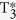, which is difficult for transferring a proton in the S_1_ state. Comparing these two kinds of excited state paths, we confirm a stepwise ESDPT mechanism for BBTD system. Under the stepwise ESDPT mechanism, the reaction pattern of BBTD system are shown (see Fig. S3, ESI† for detail). Under the pre-equilibrium among N*, 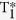 and 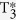, that is, K_1_, K_2_, K_3_ and K_4_ 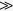 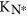, 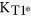 and 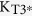 with the initial conditions as [N*]_t=0_ = 1, [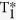] = [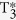]_t=0_ = 0. Accordingly, the populations of S_1_-state N*, 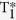 and 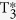 as a function of time can be obtained (shown in Fig. S4, ESI†).

### External electric field effects

**Figure 6:**
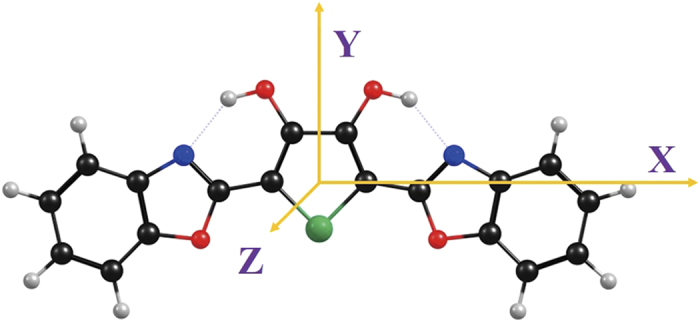
Views of three-dimensional coordinate for BBTD system.

Taking electric field effects into consideration, we apply external electric field along axis direction (see Fig. 6). The strengths of external electric field selected in this work are 5 × 10^−4^ and 10^−3^ au. For convenience, for example, by *E*_*X*_ = +5 × 10^−4^ we mean a 5 × 10^−4^ au external electric field is applied along the x axis. So the *E*_*X*_ = −5 × 10^−4^ indicates a 5 × 10^−4^ au external electric field is applied against the X axis.

**Figure 7:**
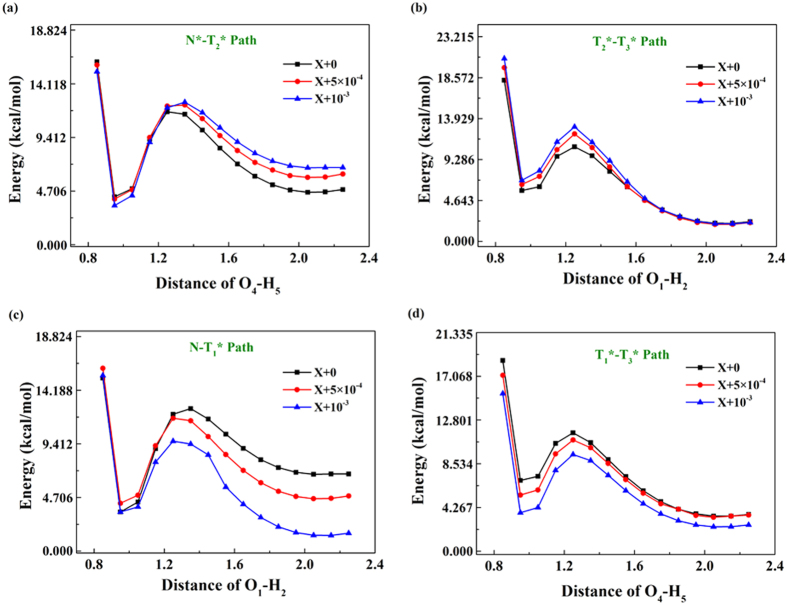
Comparing S_1_-state potential energy curves under X-axle external electric field. (**a**) The S_1_-state N*-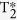 path; (**b**) the S_1_-state 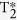-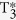 path; (**c**) the S_1_-state N*-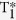 path; (**d**) the S_1_-state 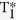-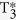 path.

To reveal differences induced by external electric field, within the framework of DFT and TDDFT B3LYP/TZVP/IEFPCM(chloroform) level, we optimize the BBTD molecule under *E*_*X*_ = +5 × 10^−4^ and *E*_*X*_ = +10^−3^. The most obvious change is the dipole moment (list in Table 2). It can be found clearly that external electric field does not have distinguishable influences on the S_0_ state, whereas it results in large changes of dipole moment in the S_1_ state. That is to say, excited state dynamical process could be largely affected by external electric field. In addition, we construct the S_0_-state and S_1_-state PESs under *E*_*X*_ = +5 × 10^−4^ and *E*_*X*_ = +10^−3^. Results show that the S_0_-state PES among no electric field (*E*_*X*_ = 0), *E*_*X*_ = +5 × 10^−4^ and *E*_*X*_ = +10^−3^ are almost the same, which confirms again that electric field has few influences on the S_0_ state. However, it is worth mentioning that external electric field changes the potential energy barriers to a great extent in the S_1_ state. Since projective plane of *E*_*X*_ = +5 × 10^−4^ or *E*_*X*_ = +10^−3^ is similar with that of normal BBTD (i.e. Fig. 5), we list primary excited-state potential barriers among these four stable points (N*, 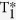, 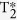 and 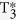) in Table 3. Also, to confirm the accuracy of B3LYP functional under external electric field, we provide the comparition between B3LYP and Cam-B3LYP functionals in Fig. S2, ESI†. It confirms the feasibility of B3LYP functional once again. In addition, the potential energy curves of characterising stepwise ESDPT under external electric field are shown in Fig. 7. Combining potential energy barriers and potential energy curves, it is obvious that the excited state path (N*-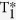-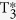) changes to be more easily along with increase of X-axle external electric field, while the second S_1_-state path (N*-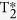-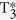) becomes more difficult to occur. Even though barrier from point N* to point 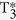 is depressed, it is still a infeasible excited state path compared to others. In addition, to aviod the situation that the changes of the potential barriers are caused by the error of the theoretical method, we also increase the external electric field to *E*_*X*_ = +3 × 10^−3^ and *E*_*X*_ = +5 × 10^−3^ (shown in Figs S5 and S6, ESI†). It can be clearly found that the enlarged external electric field do result in reduction of potential barrier for N*-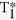-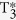 path and enlargement of potential barrier for N*-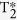-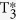 path. And even though potential barriers along with N*-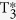 path also decrease, the extent of reduction is too small to have sufficient impact. Accordingly, we theoretically confirm that external electric field along the X axis indeed plays a part in regulating and controlling stepwise ESDPT process for BBTD system.

Similarly, we also study the external electric field along with Y axle and Z axle (i.e. *E*_*Y*_ = +5 × 10^−4^ and *E*_*Z*_ = +5 × 10^−4^). Results demonstrate that the dipole moments for BBTD in both S_0_ and S_1_ states are almost no changes, which implies that Y-axle and Z-axle external electric fields do not have obvious influences on BBTD in the S_1_ state. Indeed, we confirm this viewpoint based on constructing S_1_-state potential energy curves among four stable points. Our theoretical results show that all the S_1_-state potential energy curves are almost superposed for no external electric field, *E*_*Y*_ = +5 × 10^−4^ and *E*_*Z*_ = +5 × 10^−4^. In fact, it is worth noticing that Y-axle or Z axle external electric field is perpendicular to ESIPT orientation (see Fig. 6), while X-axle external electric field is almost parallel to the direction of ESIPT reaction. It further explains why X-axle external electric field does have obvious effects on ESDPT reaction but Y-axle or Z axle external electric field does not.

## Conclusions

In this work, within the framework of DFT and TDDFT methods, we theoretically investigate excited state dynamical process of BBTD system. Based on photoexcitation, changes about intramolecular hydrogen bonds (O_1_-H_2_ 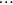 N_3_ and O_4_-H_5_ 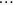 N_6_) and charge redistribution indicate tendency of ESDPT reaction. Analysis about potential energy barriers in the S_1_-state PES of BBTD reveals a stepwise ESDPT process. Considering electric field effect, we present a new mechanism about controlling S_1_-state stepwise double proton transfer path via external electric field for the first time. Herein, we sincerely wish our work can facilitate researchers to have a deeper understanding about excited state dynamical process and to pave the way for revealing new features of excited state dynamics brought by field effects.

## Additional Information

**How to cite this article**: Zhao, J. and Zheng, Y. Elaboration and controlling excited state double proton transfer mechanism of 2,5-bis(benzoxazol-2-yl)thiophene-3,4-diol. *Sci. Rep.* **7**, 44897; doi: 10.1038/srep44897 (2017).

**Publisher's note:** Springer Nature remains neutral with regard to jurisdictional claims in published maps and institutional affiliations.

## Supplementary Material

Supplementary Files

## Figures and Tables

**Table 1:** The primary bond lengths (Å) and bond angles *δ* (°) of BBTD, BBTD-SPT and BBTD-DPT structures in chloroform solvent.

	BBTD		BBTD-SPT		BBTD-DPT	
	S_0_	S_1_	S_0_	S_1_	S_0_	S_1_
O_1_-H_2_	0.986	0.992	1.983	2.088	1.993	2.101
H_2_-N_3_	1.894	1.886	1.021	1.018	1.019	1.014
O_4_-H_5_	0.986	0.992	0.985	0.990	1.993	2.101
H_5_-N_6_	1.894	1.886	1.905	1.891	1.019	1.014
*δ*(O_1_-H_2_-N_3_)	142.7	144.1	126.0	123.2	125.3	123.5
*δ*(O_4_-H_5_-N_6_)	142.7	144.1	124.3	144.4	125.3	123.5

**Table 2:** Changes of dipole moment (D) for BBTD molecule under *E*_*X*_ = 0, *E*_*X*_ = +5 × 10^−4^ and *E*_*X*_ = +10^−3^ in both S_0_ and S_1_ states.

	0	+5 × 10^−4^	+10^−3^
S_0_	3.937	4.054	4.286
S_1_	3.943	5.452	6.792

**Table 3:** Potential barriers (kcal/mol) among four stable points (N*, 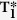 , 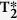 and 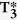 ) of BBTD molecule under *E*_*X*_ = 0, *E*_*X*_ = +5 × 10^−4^ and *E*_*X*_ = +10^−3^ on S_1_-state PESs.

	0	+5 × 10^−4^	+10^−3^
N*- 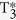	14.3	14.1	13.5
N*- 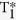	7.21	6.63	6.01
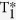 - 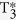	4.58	3.73	3.14
N*- 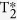	7.21	7.98	8.77
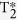 - 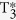	4.58	5.29	5.61
