# Supplementary material for: Elaboration and controlling excited state double proton transfer mechanism of 2,5-bis(benzoxazol-2-yl)thiophene-3,4-diol
Source: Sci Rep. 2017 Mar 22;7:44897. doi: 10.1038/srep44897 (PMC5361162; doi:10.1038/srep44897)
Supplement: Supplementary Files [file srep44897-s1.pdf]

(Submitted to Scientific Reports)

Electronic Supplementary Information (ESI)

Supplemental Material for "Elaboration and controlling excited state double proton transfer mechanism of 2, 5-bis(benzoxazol-2-yl)thiophene-3, 4-diol"

Jinfeng Zhao, Yujun Zheng*

School of Physics, Shandong University, Jinan 250100, China

E-mail address: yzheng@sdu.edu.cn

In this supplemental material, we provide in detail the simulations data, the IR spectra and the stepwise ESDPT reaction pattern.

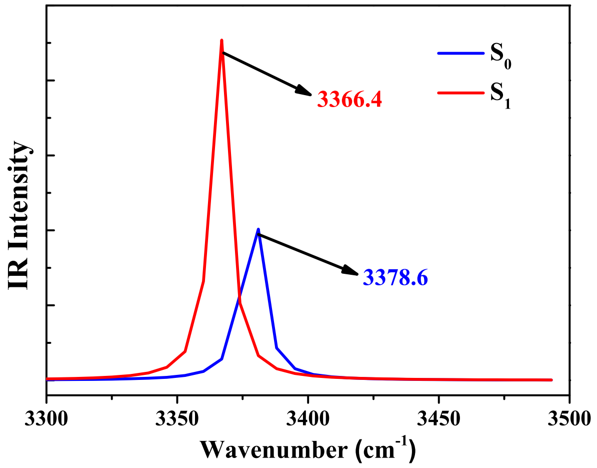

Figure S1: The theoretical IR spectra of BBTD molecule in the spectral region of both O_1-H_2 and O_4-H_5 stretching bonds in both S_0 and S_1 states.

Table S1: The thermal correction to Gibbs free energies (Hartree) of the stable BBTD, BBTD-SPT and BBTD-DPT structures in both S_0 and S_1 states.

	BBTD	BBTD-SPT	BBTD-DPT
S_0	0.1986	0.1981	0.1972
S_1	0.1950	0.1951	0.1950

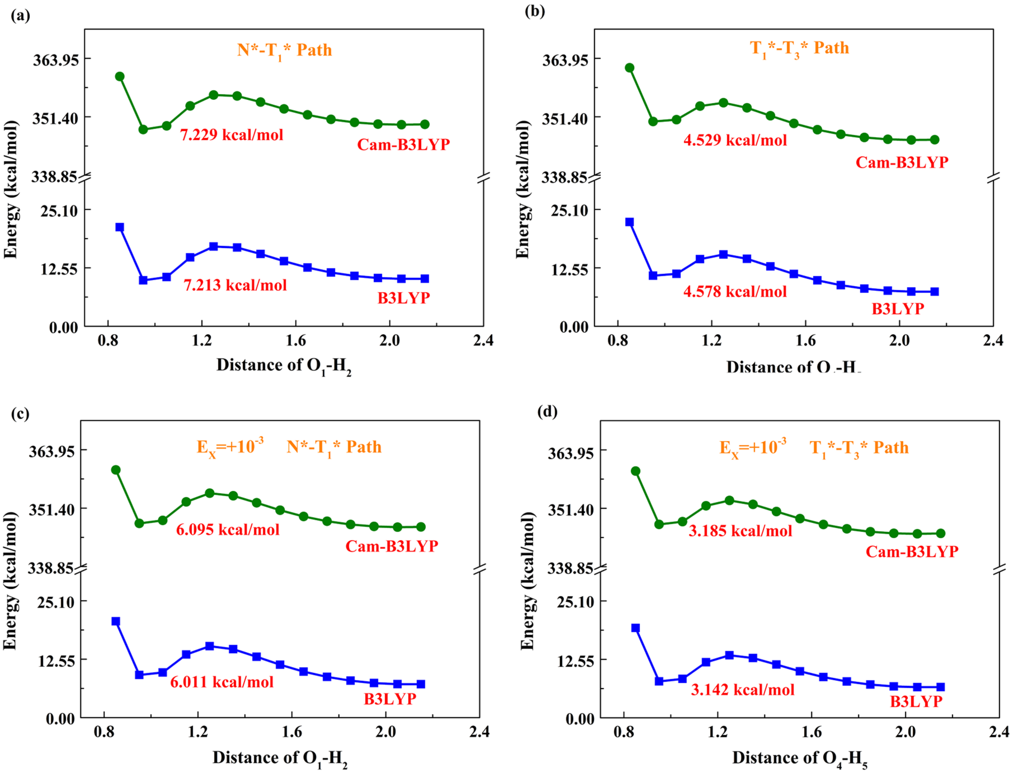

Figure S2: Comparison the PES results between B3LYP and Cam-B3LYP functionals along with N*-T_1*-T_3* path in the S_1 state. (a): the S_1-state N*-T_1* path without external electric field; (b): the S_1-state T_1*-T_3* path without external electric field; (c) the S_1-state N*-T_1* path under $E_x = +10^{-3}$; (d) the S_1-state N*-T_1* path under $E_x = +10^{-3}$.

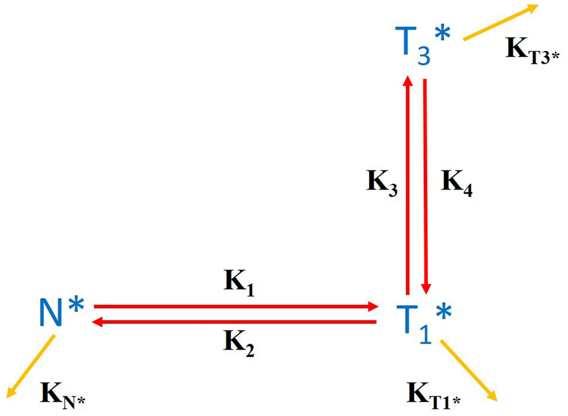

Figure S3: The stepwise ESDPT reaction pattern for BBTD system.

Herein, K_{N^*}, K_{T1^*} and K_{T3^*} mean the kinetic rate constant of both emissive and non-emissive conversion to the ground state for N*, T_1* and T_3*, respectively. K_1 and K_2 stand for the kinetic rate constant from N* to T_1* and T_1* to N*, respectively. In a similar way, K_3 and K_4 mean kinetic rate constant from T_1* to T_3* and from T_3* to T_1*, respectively. Therefore, the kinetic rate equation can be expressed as follow:

$d[N^*]/dt=-(K_{N^*}+K_1)[N^*]+K_2[T_1^*]$,

$d[T_1^*]/dt=-(K_{T1^*}+K_2+K_3)[T_1^*]+K_1[N^*]+K_4[T_3^*]$,

$d[T_3^*]/dt=-(K_{T3^*}+K_4)[T_3^*]+K_3[T_1^*]$,

where [N*], [T_1*] and [T_3*] mean the concentration of N*, T_1* and T_3*, respectively. Till now, we can show the kinetic rate equation based on the stepwise ESDPT mechanism. In fact, since all the kinetic rate constants are not provided in previous experiment, we can just provide approximate results. Herein, we take the initial conditions as $[N^*]_{t=0}=1$, $[T_1^*]=[T_3^*]_{t=0}=0$ with the assumption of K_1, K_2, K_3 and $K_4 \gg K_{N^*}$, K_{T1^*} and K_{T3^*}.

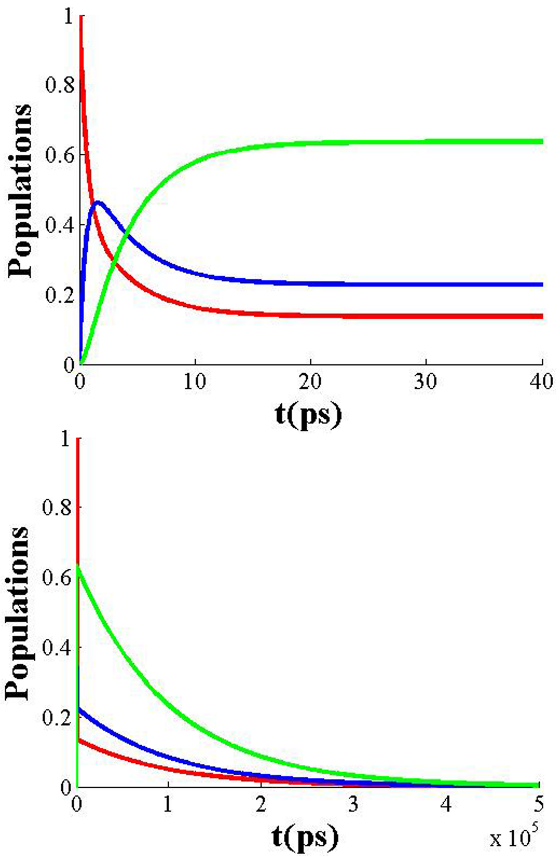

Figure S4: The evolution of populations in the S_1 state for [N*] (red line), [T_1*] (blue line) and [T_3*] (green line) as a function of time.

Clearly, stating from around 10 ps, [N*], [T_1*] and [T_3*] approximatively achieve a relative balance. Due to K_1, K_2, K_3 and $K_4 \gg K_{N^*}$, K_{T1^*} and K_{T3^*}, [N*], [T_1*] and [T_3*] would decay to zero after long time evolution (around 4×10^5 ps).

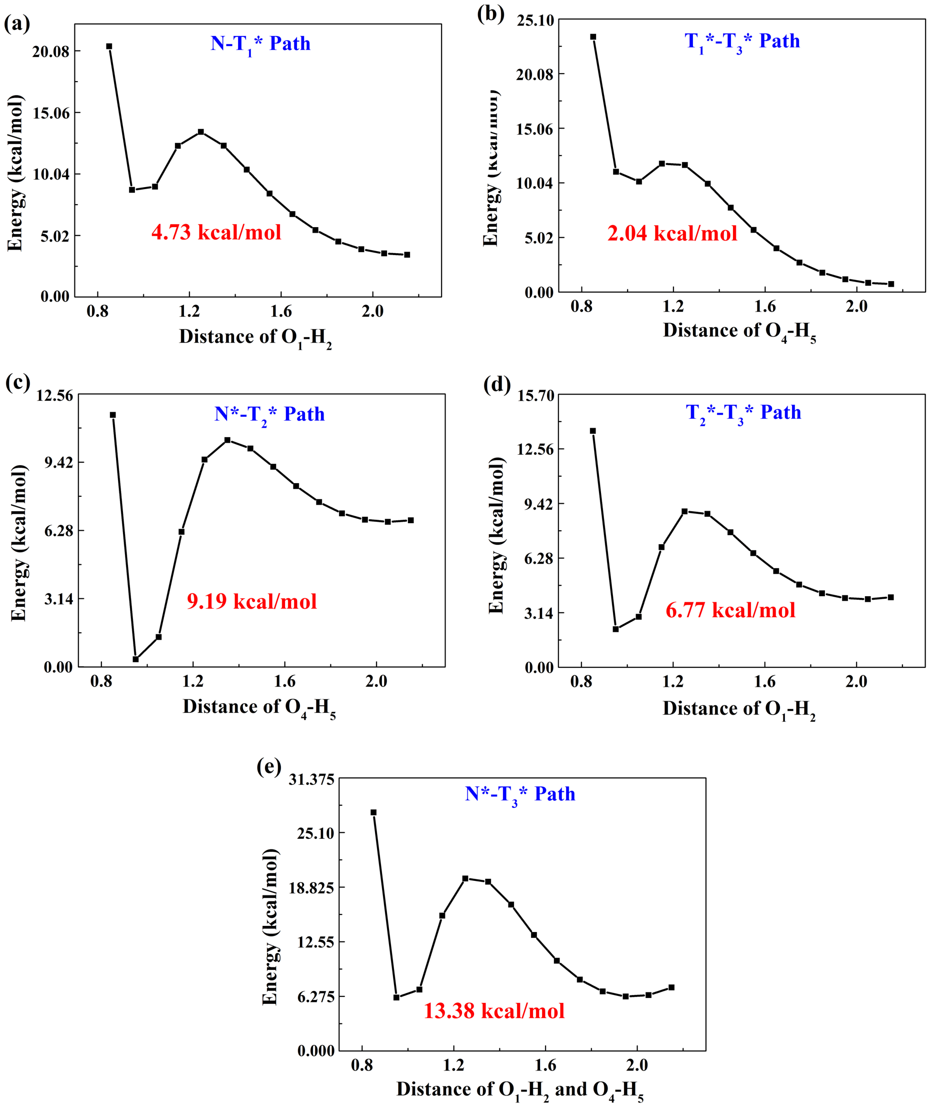

Figure S5: The S_1-state potential energy curves under $E_X = +3 \times 10^{-3}$. (a): the S_1-state N*-T_1* path; (b): the S_1-state T_1*-T_3* path; (c): the S_1-state N*-T_2* path; (d): the S_1-state T_2*-T_3* path; (e): the S_1-state N*-T_3* path. The potential barriers under these five paths are marked in red in homologous figures.

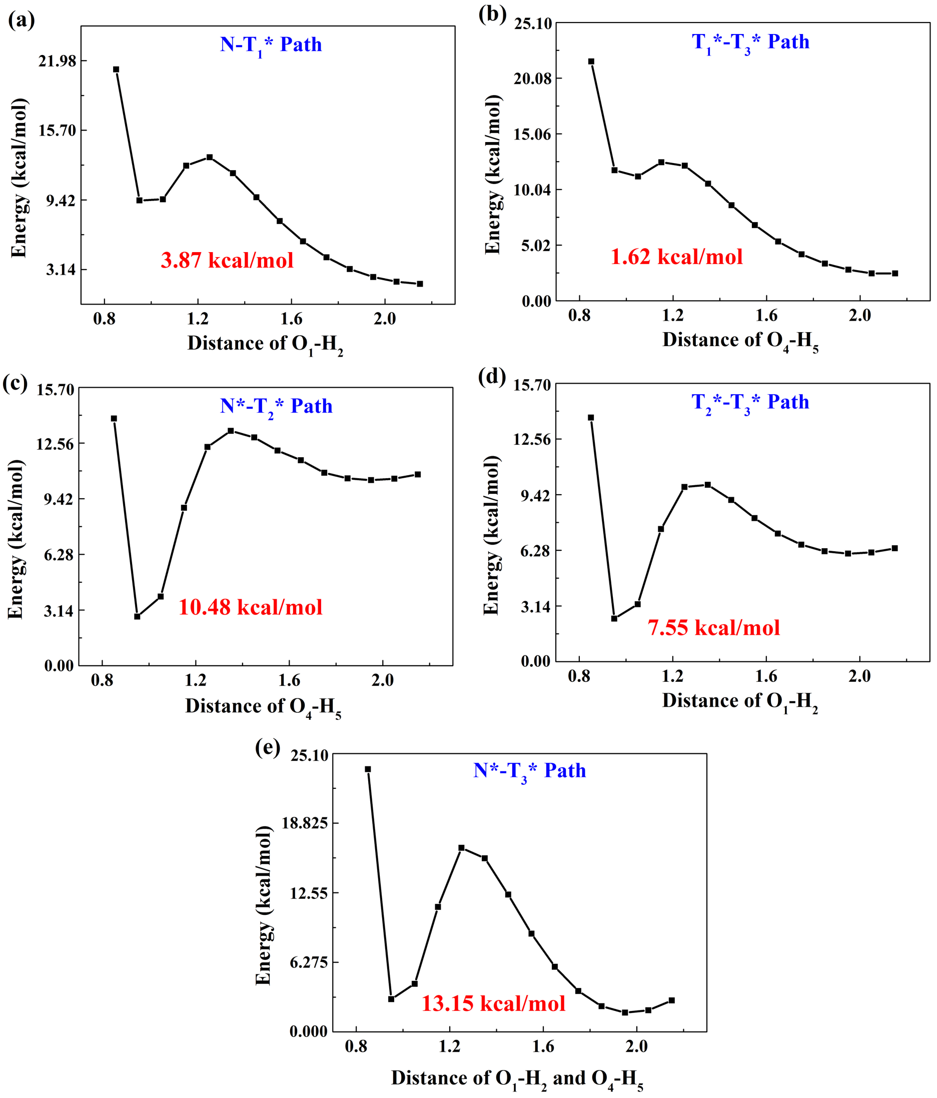

Figure S6: The S$_1$-state potential energy curves under $E_X = +5 \times 10^{-3}$. (a): the S$_1$-state N*-T$_1$* path; (b): the S$_1$-state T$_1$*-T$_3$* path; (c): the S$_1$-state N*-T$_2$* path; (d): the S$_1$-state T$_2$*-T$_3$* path; (e): the S$_1$-state N*-T$_3$* path. The potential barriers under these five paths are marked in red in homologous figures.
